# A Combined Method for MEMS Gyroscope Error Compensation Using a Long Short-Term Memory Network and Kalman Filter in Random Vibration Environments

**DOI:** 10.3390/s21041181

**Published:** 2021-02-08

**Authors:** Chenhao Zhu, Sheng Cai, Yifan Yang, Wei Xu, Honghai Shen, Hairong Chu

**Affiliations:** 1 Changchun Institute of Optics, Fine Mechanics and Physics, Chinese Academy of Sciences, Changchun 130033, China; zhuchenhao17@mails.ucas.ac.cn (C.Z.); caisheng@ciomp.ac.cn (S.C.); yangyifan17@mails.ucas.ac.cn (Y.Y.); xuweihit@ciomp.ac.cn (W.X.); 2 University of Chinese Academy of Sciences, Beijing 100049, China; 3 Key Laboratory of Airborne Optical Imaging and Measurement, Changchun Institute of Optics, Fine Mechanics and Physics, Chinese Academy of Sciences, Changchun 130033, China; shenhh@ciomp.ac.cn

**Keywords:** MEMS gyroscope, random vibration environments, long short-term memory network, Kalman filter, expectation-maximization algorithm

## Abstract

In applications such as carrier attitude control and mobile device navigation, a micro-electro-mechanical-system (MEMS) gyroscope will inevitably be affected by random vibration, which significantly affects the performance of the MEMS gyroscope. In order to solve the degradation of MEMS gyroscope performance in random vibration environments, in this paper, a combined method of a long short-term memory (LSTM) network and Kalman filter (KF) is proposed for error compensation, where Kalman filter parameters are iteratively optimized using the Kalman smoother and expectation-maximization (EM) algorithm. In order to verify the effectiveness of the proposed method, we performed a linear random vibration test to acquire MEMS gyroscope data. Subsequently, an analysis of the effects of input data step size and network topology on gyroscope error compensation performance is presented. Furthermore, the autoregressive moving average-Kalman filter (ARMA-KF) model, which is commonly used in gyroscope error compensation, was also combined with the LSTM network as a comparison method. The results show that, for the *x*-axis data, the proposed combined method reduces the standard deviation (STD) by 51.58% and 31.92% compared to the bidirectional LSTM (BiLSTM) network, and EM-KF method, respectively. For the *z*-axis data, the proposed combined method reduces the standard deviation by 29.19% and 12.75% compared to the BiLSTM network and EM-KF method, respectively. Furthermore, for *x*-axis data and *z*-axis data, the proposed combined method reduces the standard deviation by 46.54% and 22.30% compared to the BiLSTM-ARMA-KF method, respectively, and the output is smoother, proving the effectiveness of the proposed method.

## 1. Introduction

Fiber optic gyroscopes and laser gyroscopes have excellent performance, but they are too large and expensive for portable devices [1,2]. Micro-electro-mechanical-system (MEMS) gyroscopes have, in recent years, been used in low-cost inertial navigation systems (INS) due to their small size and low cost. However, the MEMS gyroscope has a significant error due to the manufacturing technology and structural composition [3,4]. The error of the MEMS gyroscope can be divided into deterministic error and random error. The deterministic error mainly refers to perturbation errors such as zero offsets and the scale factor, which can be corrected by a calibration test [5,6]. Random error refers to the random drift caused by uncertain factors, usually determined by the device’s accuracy level [7], with no precise repeatability. Therefore, it is difficult to accurately compensate for random error, which hinders the further improvement of MEMS gyroscope performance.

In MEMS gyroscope error compensation research, the MEMS gyroscope data are generally treated as time-series data. Scholars have proposed methods such as the autoregressive moving average (ARMA) model, the Allan variance (AV), the wavelet threshold (WT), the support vector machine (SVM), and the artificial neural network (ANN), and all of them have achieved excellent results [7,8,9,10,11,12,13,14]. Recently, various variants of the recurrent neural network (RNN), which has strong processing power for time-series data, have been shown to be superior to traditional methods in the research of error compensation in MEMS gyroscopes [15,16,17,18].

However, most of the research mentioned above has acquired data by placing the MEMS gyroscope in a static environment. In practical applications of the MEMS gyroscope, it is inevitably that it is affected by random vibration [19]. In random vibration environments, the MEMS gyroscope is interfered with by both internal device noise and external vibration noise [20], which dramatically affects the performance of the MEMS gyroscope. The degradation of performance in vibrating environments is a fatal problem for MEMS gyroscopes [21,22], so it is essential to research error compensation methods in random vibration environments.

Most of the current research on improving the performance of the MEMS gyroscope in random vibration environments is to fix the MEMS gyroscope on a vibration isolation platform [23,24,25]. However, this kind of method is not universal [26]. There is not much research based on time-series models—the windowed measurement error covariance (WMEC) method has been applied to compensate for the effects of the vibration environments [27], singular spectrum analysis (SSA) was proposed to remove the low-frequency vibration noise perturbations of MEMS accelerometers [28], and the third-order autoregressive (AR) model was used to estimate the Kalman filter to compensate for the MEMS gyroscope’s attitude angle error caused by random vibration [29].

Considering the dramatic perturbation of the MEMS gyroscope in random vibration environments, in this paper, a combined method of a long short-term memory (LSTM) network and Kalman filter is proposed for error compensation, with the Kalman smoother and expectation-maximization (EM) algorithm to dynamically adjust the predicted values of the LSTM network to improve the performance in error compensation. The main contributions of this paper are as follows:

(1) The combination of LSTM network and Kalman filter is applied to MEMS gyroscope error compensation in random vibration environments;
(2) The proper input data step and the network topology are explored, and the error compensation performance of the bidirectional LSTM (BiLSTM) network and other recurrent neural network (RNN) variants are compared;
(3) In designing the Kalman filter, the EM algorithm is used to estimate the parameters. It is compared with the ARMA model, a parameter estimation method commonly used in research of the MEMS gyroscope error compensation problem.

The remainder of this paper is organized as follows: (1) Section 2 introduces the methods, including BiLSTM network, Kalman filter, ARMA-KF model, and EM-KF model, and gives the illustration of this paper proposed method; (2) Section 3 presents the experiment, results, and comparisons; and (3) the remaining sections are the conclusion, appendix, and references.

## 2. Method

### 2.1. Multi-Layer BiLSTM Network and Kalman Filter

The long short-term memory network is a variant of the recurrent neural network used to solve the gradient vanishing or gradient explosion problem of RNNs [30,31]. A detailed description of LSTM units can be found in references [15,16,17,18].

**Figure 1:**
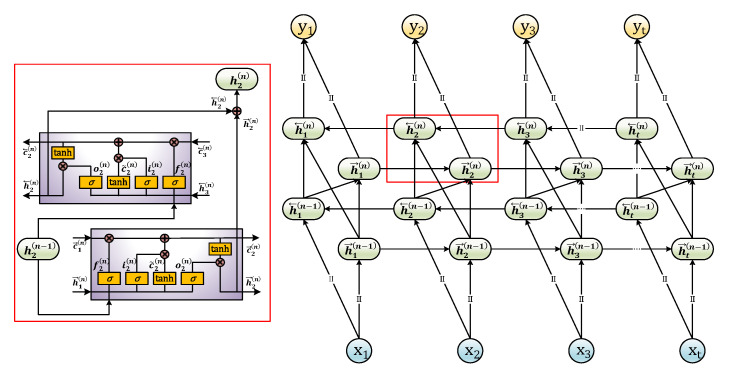
Information flow of multi-layer bidirectional long short-term memory (BiLSTM) network.

The basic LSTM network only considers the historical and current inputs and ignores future inputs [32]. Therefore, the LSTM network can perform the reverse operation, superimpose the forward and reverse information flows, and fully utilize the front and back inputs at the current time to improve the error compensation performance. In addition, the previous hidden layer’s output is used as the input of the following layer to explore the more in-depth features of the time-series data, thus enhancing the model’s nonlinear fitting ability. The multi-layer BiLSTM network information flow is shown in Figure 1.

The cell state of the Layer *n* BiLSTM network at time *t* can be presented as:

(1) $$\left[\begin{array}{c} i_{t}^{\left(n\right)}\\ f_{t}^{\left(n\right)}\\ o_{t}^{\left(n\right)}\\ {\tilde{c}}_{t}^{\left(n\right)} \end{array}\right]=[\begin{array}{c} \sigma \\ \sigma \\ \sigma \\ tanh \end{array}]\;\left[\begin{array}{cc} W_{i,x}^{\left(n\right)} & W_{i,h}^{\left(n\right)}\\ W_{f,x}^{\left(n\right)} & W_{f,h}^{\left(n\right)}\\ W_{o,x}^{\left(n\right)} & W_{o,h}^{\left(n\right)}\\ W_{\tilde{c},x}^{\left(n\right)} & W_{\tilde{c},h}^{\left(n\right)} \end{array}\right]\;\left[\begin{array}{c} h_{t}^{\left(n-1\right)}\\ h_{t}^{\left(n\right)} \end{array}\right]$$

where $h_{t}^{\left(n-1\right)}$ is the hidden state of the Layer n−1 at time t. Each hidden state is composed of forward and reverse superposition. The related equations are denoted as follows:

(2) $$h_{t}^{\left(n\right)}={\vec{h}}_{t}^{\left(n\right)}⊕{\overset{\leftarrow }{h}}_{t}^{\left(n\right)}$$

The Kalman filter is an optimal state estimation method that can be applied to dynamic systems with random disturbances. It estimates the system state based on discrete measurement that contain noise [33,34]. Suppose the state–space model is built as:

(3) $${\hat{x}}_{k}=\Phi {\hat{x}}_{k-1}+\Gamma \omega_{k-1}$$

(4) $$y_{k}=Hx_{k}+v_{k}$$

where Φ is the system state transition matrix, Γ is the system noise-driven matrix, H is the measurement matrix, ${\hat{x}}_{k}$ is the system state vector, $y_{k}$ is the measurement vector, $\omega_{k}$ is the system noise vector, and $v_{k}$ is the measurement noise vector.

The noise of the system models and measurement models are assumed to have normal distribution in the Kalman filter, such that [35]:

(5) $$\omega_{k}∼N\left(0,Q\right)$$

(6) $$v_{k}∼N\left(0,R\right)$$

where Q is the covariance matrix of the system models and R is the covariance matrix of measurement models.

The Kalman filter is composed of two-stage optimization. In the predicted stage, the current system state vector is predicted based on the system state vector at the previous time, such that:

(7) $${\hat{x}}_{k/k-1}=\Phi {\hat{x}}_{k-1}$$

(8) $$P_{k/k-1}=\Phi P_{k-1}\Phi^{T}+\Gamma Q\Gamma^{T}$$

where ${\hat{x}}_{k/k-1}$ is the predicted value of the system state vector and $P_{k/k-1}$ is the predicted covariance matrix of the system state vector.

In the updated stage of the Kalman filter, the current system state vector is updated by using the measurement vector, such that:

(9) $$K_{k}=P_{k/k-1}H^{T}{\left(HP_{k/k-1}H^{T}+R\right)}^{-1}$$

(10) $${\hat{x}}_{k}={\hat{x}}_{k/k-1}+K_{k}\left(y_{k}-H{\hat{x}}_{k/k-1}\right)$$

(11) $$P_{k}=\left(I-K_{k}H\right)P_{k/k-1}$$

where $K_{k}$ is the Kalman filter gain matrix and $P_{k}$ is the updated covariance matrix of the system state vector.

### 2.2. Kalman Filter Design with ARMA Model

The autoregressive moving average model is the most widespread model used in time-series analysis, and it is derived and developed on the basis of the linear regression model. The ARMA model can be described as [36]:

(12) $$x_{t}=\overset{p}{\underset{i=1}{\sum }}\varphi_{i}x_{t-i}-\overset{q}{\underset{j=1}{\sum }}\theta_{j}\varepsilon_{t-j}+\varepsilon_{t},\;\varepsilon_{t}∼N\left(0,\delta_{\varepsilon }^{2}\right)$$

where p and q are the order of the ARMA model; $\varphi_{i}$ and $\theta_{j}$ are coefficients that satisfy stationary and invertible conditions, respectively [37]; $\mathrm{and}\;\varepsilon_{t}$ is white noise, which is an uncorrelated random variable with mean zero and constant variance. The model expresses that the measured values of the stochastic process $\left\{x_{t}\right\}$ at time t are correlated with the previous p measurements and the previous q white noise.

The steps to design a Kalman filter using the ARMA model as follows: (1) test the stationarity and normality of the measurement data, (2) determine the model type according to the autocorrelation function and partial autocorrelation function, (3) determine the order and parameters of the model according to the Akaike information criterion (AIC) [38], and (4) perform adaptive testing of the designed model.

### 2.3. Kalman Filter Design with EM Algorithm

The expectation-maximization algorithm is an iterative method proposed by Shumway and Stoffer to compute maximum likelihood estimates based on incomplete data [39]. It is convergent and can identify parameters and states in the model [40]. Andrieu and Doucet introduced the EM algorithm for parameter estimation for linear state–space models was introduced [41]. The EM algorithm is an iterative numerical algorithm for computing the maximum likelihood estimation (MLE). The linear Gaussian state–space model used for the EM algorithm can be expressed as follows [42]:

(13) $$x_{k}=\Phi x_{k-1}+\omega_{k-1}$$

(14) $$y_{k}=Hx_{k}+\upsilon_{k}$$

The conditional probability densities of the state equation and the measurement equation are obtained from Equations (13) and (14), respectively:

(15) $$p\left(x_{k}|x_{k-1}\right)=\exp\left\{-\frac{1}{2}{\left[x_{k}-\Phi x_{k-1}\right]}^{T}Q^{-1}\left[x_{k}-\Phi x_{k-1}\right]\right\}{\left(2\pi \right)}^{-\frac{n}{2}}{\left|Q\right|}^{-\frac{1}{2}}$$

(16) $$p\left(y_{k}|x_{k}\right)=\exp\left\{-\frac{1}{2}{\left[y_{k}-Hx_{k}\right]}^{T}R^{-1}\left[y_{k}-Hx_{k}\right]\right\}{\left(2\pi \right)}^{-\frac{m}{2}}{\left|R\right|}^{-\frac{1}{2}}$$

It is assumed that the likelihood of the system state data and the evolution of the states is Gaussian, which are defined by the following equations [43]:

(17) $$p\left(Y,X|\Theta \right)=p\left(x_{1}\right)\overset{N}{\underset{k=1}{\prod }}p\left(y_{k}|x_{k}\right)\overset{N}{\underset{k=2}{\prod }}p\left(x_{k}|x_{k-1}\right)$$

where Y is the measurement data, X is the unknown system state data, and Θ is the parameter set of linear Gaussian state–space model. Θ can be represented as follows:

(18) Θ={Φ,H,Q,R}

By taking the log of the likelihood we arrive at the following formula:

(19) $$\ln p\left(Y,X|\Theta \right)=-\frac{1}{2}\overset{N}{\underset{k=2}{\sum }}\left\{\ln\left|Q\right|+{\left[x_{k}-\Phi x_{k-1}\right]}^{T}Q^{-1}\left[x_{k}-\Phi x_{k-1}\right]\right\}-\frac{1}{2}\overset{N}{\underset{k=1}{\sum }}\left\{\ln\left|R\right|+{\left[y_{k}-Hx_{k}\right]}^{T}R^{-1}\left[y_{k}-Hx_{k}\right]\right\}$$

Depending on the maximum likelihood method, the linear Gaussian state–space model can be identified through an EM algorithm [42]. The algorithm alternates between two steps—the E-step (expectation) and the M-step (maximization) [44]. In general, the likelihood density function based on the measurement data, denoted by p(Θ|Y), is called the posterior distribution of the measurement. The EM algorithm aims to compute the maximum likelihood estimation of p(Θ|Y). $\Theta_{i}$ is denoted as the estimate of the likelihood function at the beginning of the ith iteration.

In the E-step, the expectation for the conditional distribution of lnp(Y,X|Θ) concerning X, is calculated such that:

(20) $$\Omega \left(\Theta |\Theta_{i},Y\right)≙E_{X}\left\{\ln p\left(\Theta |Y,X\right)|\Theta_{i},Y\right\}=\int \left[\ln p\left(\Theta |Y,X\right)\right]\left(X|\Theta_{i},Y\right)dX$$

In the M-step, $\Omega \left(\Theta |\Theta_{i},Y\right)$ is maximized to find $\Theta_{i+1}$ such that:

(21) $$\Omega \left(\Theta_{i+1}|\Theta_{i},Y\right)=arg\underset{\Theta }{\max}\left[\Omega \left(\Theta |\Theta_{i},Y\right)\right]$$

The E-step and the M-step are iterated until,

(22) $$\|L\left(\Theta_{i+1}\right)-L\left(\Theta_{i}\right)\|<\tau$$

where τ is the predefined threshold. Equation (22) means that it has satisfied the convergence criterion. The specific process of designing a Kalman filter using the EM algorithm is given as follows:

The value of $\Omega \left(\Theta |\Theta_{i},Y\right)$ is determined by the following [45]:

(23) $$E_{X}\left(x_{k}|Y\right)={\hat{x}}_{k|N}$$

(24) $$E_{X}\left(x_{k}x_{k-1}^{T}|Y\right)=P_{k,k-1|N}+{\hat{x}}_{k|N}{\hat{x}}_{k-1|N}^{T}$$

(25) $$E_{X}\left(x_{k}x_{k}^{T}|Y\right)=P_{k|N}+{\hat{x}}_{k|N}{\hat{x}}_{k|N}^{T}$$

where ${\hat{x}}_{k|N}$ is the smoothed value of the system state vector and $P_{k|N}$ is the smoothed covariance matrix of the system state vector. $P_{k,k-1|N}$ is initialized by:

(26) $$P_{k,k-1|k}=\left(I-K_{k}H\right)\Phi P_{k-1}$$

(27) $$P_{k,k-1|N}=P_{k,k-1|k}+\left[P_{k|N}-P_{k}\right]P_{k|k}^{-1}P_{k,k-1|k}$$

${\hat{x}}_{k|N}$ and $P_{k|N}$ can be obtained by smoothing the outputs of Kalman filter using backward-pass methods such as the Rauch–Tung–Striebel (RTS) smoother [46]. This method is summarized in the following equations:

(28) $$J_{k-1}=P_{k-1}\Phi^{T}P_{k|k-1}^{-1}$$

(29) $${\hat{x}}_{k-1|N}={\hat{x}}_{k-1}+J_{k-1}\left({\hat{x}}_{k|N}-{\hat{x}}_{k/k-1}\right)$$

(30) $$P_{k-1|N}=P_{k-1}-J_{k-1}\left(P_{k|N}-P_{k|k-1}\right)J_{k-1}^{T}$$

Then, the model parameters are re-estimated by maximizing the $\Omega \left(\Theta |\Theta_{i},Y\right)$ over Θ using partial derivatives of $\Omega \left(\Theta |\Theta_{i},Y\right)$ and setting them to zero. Solving these equations yields the updated parameters (in the ith iteration) as follows:

(31) $$\frac{\partial L\left(\Theta \right)}{\partial \Phi }=-\overset{N}{\underset{k=2}{\sum }}Q^{-1}\left(P_{k,k-1|N}+{\hat{x}}_{k|N}{\hat{x}}_{k|N}^{T}\right)+\overset{N}{\underset{k=2}{\sum }}Q^{-1}\Phi \left(P_{k-1|N}+{\hat{x}}_{k-1|N}{\hat{x}}_{k-1|N}^{T}\right)=0$$

(32) $$\Phi_{i+1}=\left(\overset{N}{\underset{k=2}{\sum }}P_{k,k-1|N}+{\hat{x}}_{k|N}{\hat{x}}_{k|N}^{T}\right){\left(\overset{N}{\underset{k=2}{\sum }}P_{k-1|N}+{\hat{x}}_{k-1|N}{\hat{x}}_{k-1|N}^{T}\right)}^{-1}$$

(33) $$\frac{\partial L\left(\Theta \right)}{\partial H}=-\overset{N}{\underset{k=1}{\sum }}R^{-1}y_{k}{\hat{x}}_{k|N}^{T}+\overset{N}{\underset{k=1}{\sum }}R^{-1}H\left(P_{k|N}+{\hat{x}}_{k|N}{\hat{x}}_{k|N}^{T}\right)=0$$

(34) $$H_{i+1}=\left(\overset{N}{\underset{k=1}{\sum }}y_{k}{\hat{x}}_{k|N}^{T}\right){\left[\overset{N}{\underset{k=1}{\sum }}\left(P_{k|N}+{\hat{x}}_{k|N}{\hat{x}}_{k|N}^{T}\right)\right]}^{-1}$$

(35) $$\frac{\partial L\left(\Theta \right)}{\partial Q^{-1}}=\frac{N}{2}Q-\frac{1}{2}\overset{N}{\underset{k=1}{\sum }}\left(P_{k|N}+{\hat{x}}_{k|N}{\hat{x}}_{k|N}^{T}\right)+\Phi \left[\frac{1}{N}\overset{N}{\underset{k=2}{\sum }}\left(P_{k,k-1|N}+{\hat{x}}_{k|N}{\hat{x}}_{k-1|N}^{T}\right)\right]=0$$

(36) $$Q_{i+1}=\frac{1}{N}\left(\overset{N}{\underset{k=1}{\sum }}\left(P_{k|N}+{\hat{x}}_{k|N}{\hat{x}}_{k|N}^{T}\right)-\Phi_{i+1}\overset{N}{\underset{k=2}{\sum }}\left(P_{k,k-1|N}+{\hat{x}}_{k|N}{\hat{x}}_{k-1|N}^{T}\right)\right)$$

(37) $$\frac{\partial L\left(\Theta \right)}{\partial R^{-1}}=\frac{N+1}{2}R-\overset{N}{\underset{k=1}{\sum }}\left(\frac{1}{2}y_{k}y_{k}^{T}-H{\hat{x}}_{k|N}y_{k}^{T}+\frac{1}{2}H\left(P_{k|N}+{\hat{x}}_{k|N}{\hat{x}}_{k|N}^{T}\right)H^{T}\right)=0$$

(38) $$R_{i+1}=\frac{1}{N+1}\left(\overset{N}{\underset{k=1}{\sum }}y_{k}y_{k}^{T}\right)-H_{i+1}{\left(\frac{1}{N+1}\overset{N}{\underset{k=1}{\sum }}y_{k}{\hat{x}}_{k|N}\right)}^{T}$$

**Figure 2:**
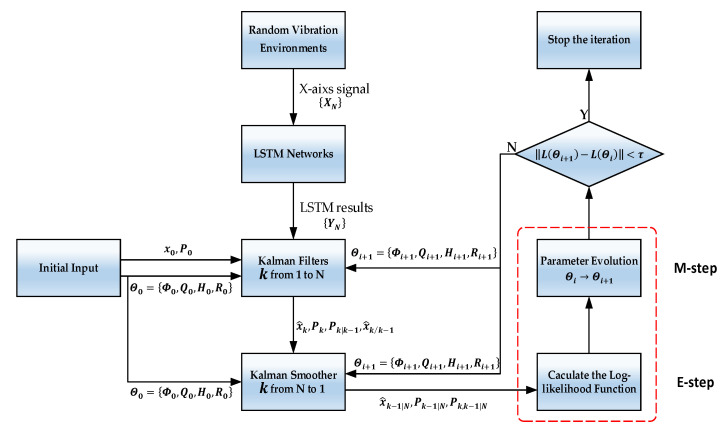
An illustration of this paper’s proposed method.

In this paper, based on the EM algorithm, the proposed LSTM and Kalman filter combination method is illustrated in Figure 2.

## 3. Experiments and Results

In this section, the designed experiments and the analysis of the results are presented to verify the effectiveness of the proposed method.

### 3.1. Data Acquisition

**Figure 3:**
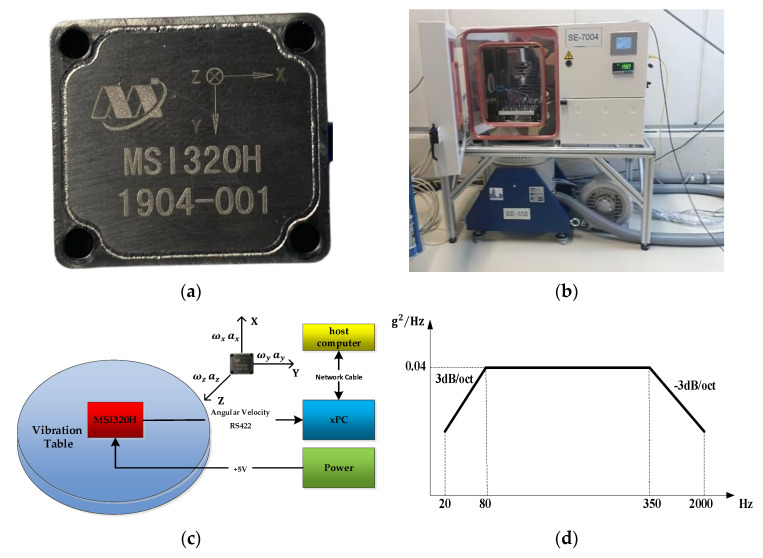
Experimental system. (**a**) MSI3200H inertial measurement unit, (**b**) vibration table, (**c**) data acquisition procedure, and (**d**) power spectral density of linear random vibration loads.

The MSI320H MEMS Inertial Measurement Unit (IMU) was employed for experiments. This consists of a three-axis MEMS gyroscope and a three-axis MEMS accelerometer. The real picture and the gyroscope specifications of MSI320H are shown in Figure 3a and Table 1, respectively. The MSI320H was fixed on the vibration table. A picture of the vibration table is shown in Figure 3b. The data acquisition procedure of the MSI320H is shown in Figure 3c. Data from the MSI320H was sent to the xPC via the RS422 communication interface with a Baud of 921,600 bps. The xPC decoded the gyroscope data and sent it to the host computer via the network cable. The MSI320H was preheated at room temperature with power for 20 minutes. Then, linear vibration experiments were performed. The vibration direction of the vibration table is the *y*-axis of the gyroscope, and the power spectral density (PSD) of the linear random vibration loads is shown in Figure 3d.

**Figure 4:**
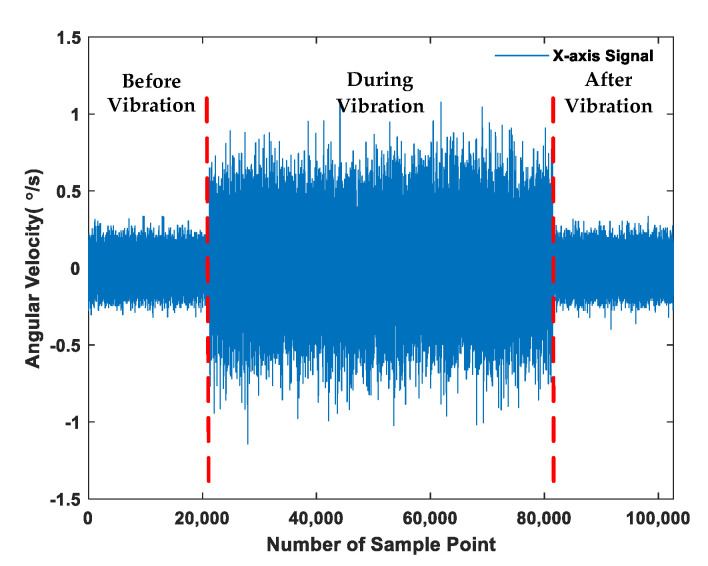
The variation of gyroscope *x*-axis signal.

As illustrated in Figure 3d, the acceleration of the applied vibration loads can be expressed as follows:

(39) $$a_{v}\left(t\right)=\xi_{v}\sin\left(\omega_{v}t\right),\;\omega_{v}\in \left[20\cdot 2\pi ,2000\cdot 2\pi \right]$$

where $\xi_{v}=6$ g. The sample rate was set to 200 Hz, and approximately 60,000 data were acquired in the random vibration environment. The variation of gyroscope *x*-axis signal affected by random vibration is shown in Figure 4, and the error of the gyroscope increased significantly in random vibration environments.

### 3.2. Comparison of BiLSTM and Other RNN Variants

The outliers of the acquired data in random vibration environments were eliminated using the Puata criterion [47]. Because the linear random vibration test’s direction was the *y*-axis of the gyroscope, the *x*-axis and *z*-axis directions were in a random vibration environment. For the consideration of model generality, we took the last 80% of the processed *x*-axis data as the training set and the first 20% of the *x*-axis and *z*-axis data were used as the testing set.

**Figure 5:**
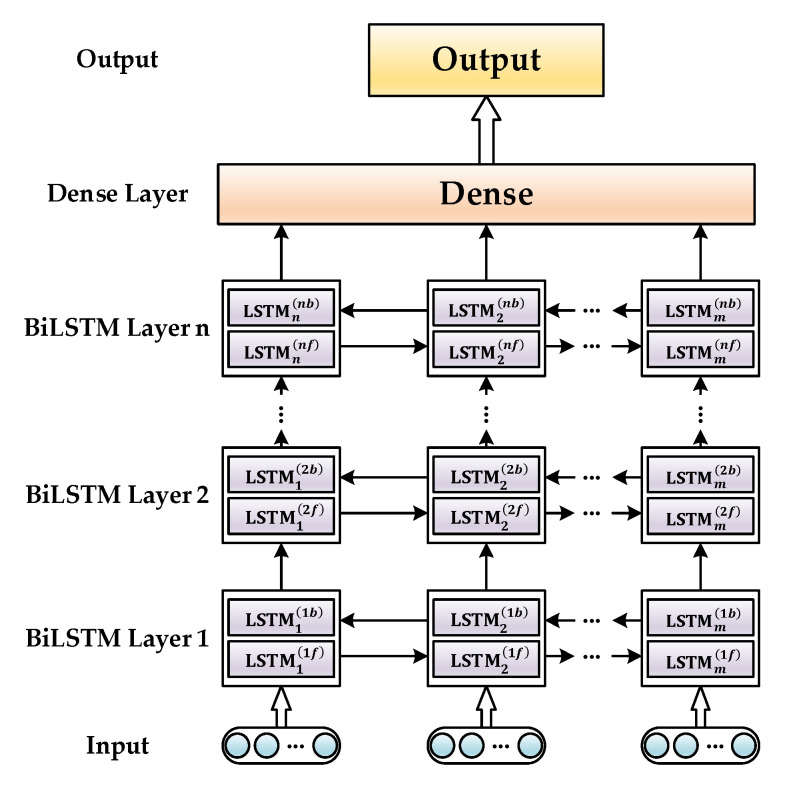
Multi-layer BiLSTM network.

If the hidden layer structure of the designed network is too simple, it is not easy to characterize the time-series model of gyroscope data. Conversely, it increases the complexity of the network, reduces the learning speed of the network, and tends to fall into local minima during the learning process. With the above considerations, in this paper, the proposed BiLSTM network is shown in Figure 5. The dense layer transformed the high-dimensional stacked sequence of the hidden layer into an output sequence with the same shape as the input sequence. Moreover, considering a large number of network parameters, dropout was set in the dense layer to prevent the overfitting phenomenon [48].

The adaptive moment estimation (Adam) optimization algorithm was used to update the network parameters [49]. The Adam algorithm uses default parameters. The activation function of the Dense layer is rectified linear unit (ReLU). The specifications used for network training are illustrated in Table 2. Moreover, the root mean square error (RMSE) was used as the loss function. The network training was performed on Tensorflow 2.0.0 and Keras 2.3.1 over the Ubuntu 16.04-LTS-x86 64 operating system. The heterogeneous computing platform was equipped with Intel Xeon E5-1620 and GeForce RTX-2080Ti GPUs.

In order to verify the performance of BiLSTM for gyroscope error compensation in random vibration environments, proper values for the input data step size, the number of hidden units, and the number of hidden layers was first explored using the *x*-axis testing set. Subsequently, training was performed using the identified values. The BiLSTM network results were compared with the LSTM network, gated recurrent unit (GRU) network, and bidirectional GRU (BiGRU) network using the *x*-axis and *z*-axis testing sets, respectively.

As shown in Table 3, Table 4 and Table 5, when the input data step size and the number of hidden layers are more extensive, the training time per epoch will be longer. So we needed to make a trade-off between the results and the computational performance. According to the results, the best results were obtained when taking the input data step of 20, the number of hidden units of 128, and the number of hidden layers of 10. Although it does not indicate that this is the optimal parameter for the network, it will be the proper value to be obtained considering the computational resources.

**Figure 6:**
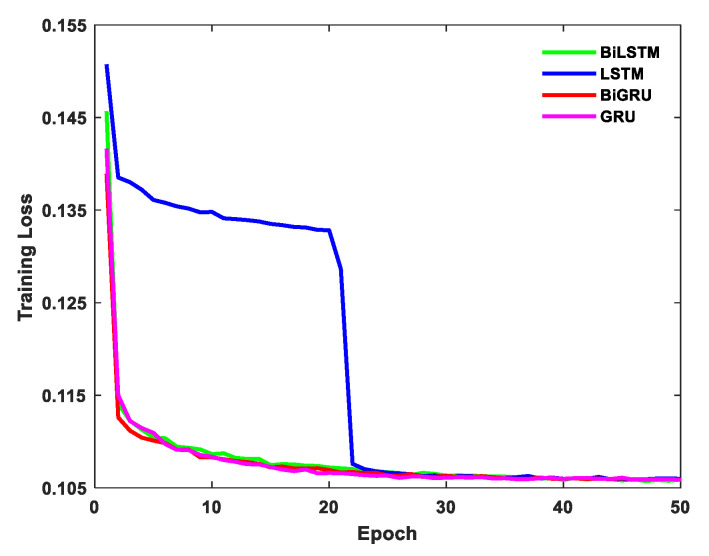
Training loss of BiLSTM, LSTM, BiGRU, and GRU.

**Figure 7:**
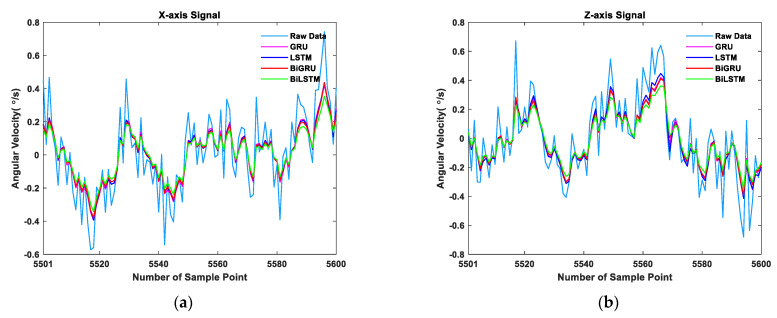
Comparison of error compensation performance of BiLSTM, LSTM, BiGRU, and GRU. (**a**) Part of the *x*-axis data zoomed-in and (**b**) part of the *z*-axis data zoomed-in.

The results are shown in Figure 6 and Figure 7 and Table 6 and Table 7. Figure 6 shows the training loss within 50 epochs, and convergence was achieved for all networks. Table 6 and Table 7 show that the standard deviations of the BiLSTM network results for the *x*-axis and *z*-axis were reduced by 46.81% and 43.63%, respectively, compared to the raw data, proving that the BiLSTM network is feasible for the application in the research of MEMS gyroscope error compensation. Furthermore, compared with the results of the LSTM network, the BiGRU network, and the GRU network, the standard deviation values of BiLSTM results in the *x*-axis were reduced by 14.06%, 11.66%, and 17.33%, respectively, and the standard deviation values of BiLSTM results in the *z*-axis were reduced by 12.71%, 10.04%, and 14.04%, respectively. This indicates that the error compensation performance of the BiLSTM network is better than these three networks.

### 3.3. Comparison of LSTM-EM-KF and LSTM-ARMA-KF

In this section, the raw data and BiLSTM network results of the *x*-axis and *z*-axis are used as the measurement, respectively. The Kalman filter parameters are estimated by the ARMA model and EM algorithm, and the filter results are compared.

#### 3.3.1. Estimating Kalman Filter Parameters Using the ARMA Model

When modeling time-series data using the ARMA model, the time-series data must meet stationarity and normality requirements. Therefore, a polynomial fitting method was used to eliminate the trend term before modeling. In this paper, the stationarity was tested using the run test, and the normality was tested by calculating the skewness, ξ, and the kurtosis, υ.

**Figure A1:**
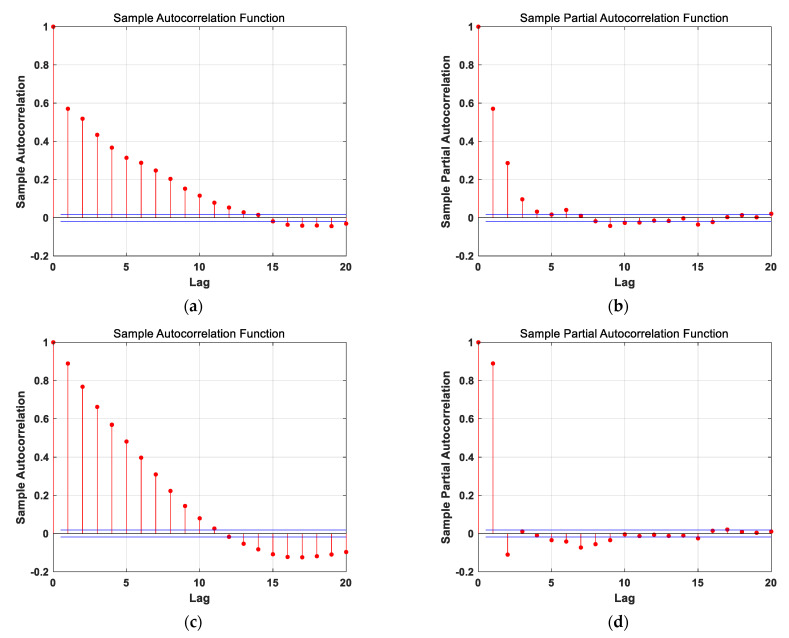

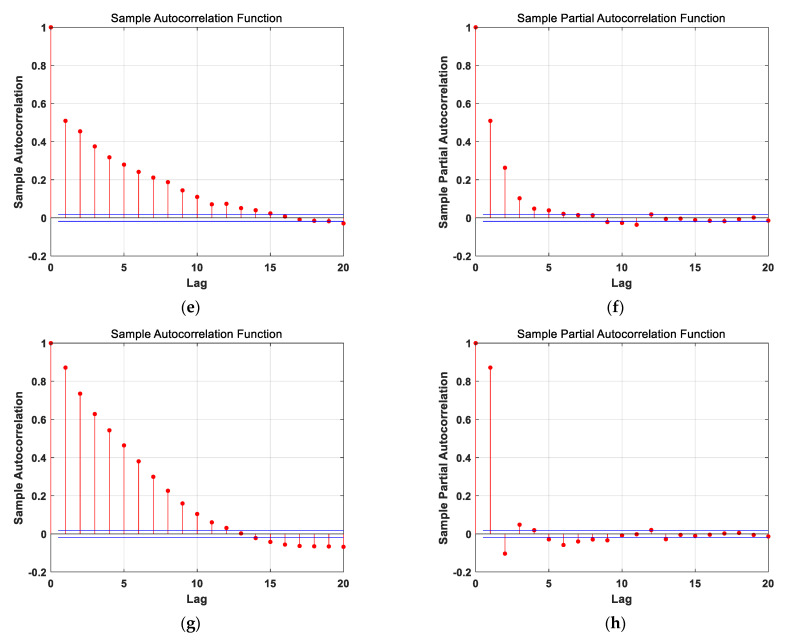
Autocorrelation function and partial autocorrelation function diagram. (**a**,**b**) *x*-axis raw data, (**c**,**d**) *x*-axis BiLSTM network results, (**e**,**f**) *z*-axis raw data, and (**g**,**h**) *z*-axis BiLSTM network results.

According to the test, after eliminating the trend term, the raw data and BiLSTM network results of the *x*-axis and *z*-axis met the stationarity and normality requirements. The test process is shown in Appendix A. Moreover, as illustrated in Figure A1, the autocorrelation function and partial autocorrelation function diagrams exhibit trailing properties, and all models can be identified as an ARMA (p,q) model.

The next step is to determine the order of the model. If the order is increased, the identified model will be more realistic, but the computational difficulty will also increase as the order increases. Therefore, the maximum order was set to 3, which means the maximum value of p and q was set to 3. Furthermore, the Akaike information criterion (AIC) was used for determining model order. Determining the model order process and the Durbin–Watson test results are shown in Appendix B.

For *x*-axis raw data, the model identified is identified as ARMA(3,3):

(40) $$x_{n}=-0.3126x_{n-1}+0.8168x_{n-2}+0.1520x_{n-3}+\varepsilon_{n}+0.6885\varepsilon_{n-1}-0.3241\varepsilon_{n-2}-0.0366\varepsilon_{n-3}$$

For *x*-axis BiLSTM network results, the model is identified as ARMA(3,3):

(41) $$x_{n}=0.8505x_{n-1}+0.8891x_{n-2}-0.7745x_{n-3}+\varepsilon_{n}+0.1354\varepsilon_{n-1}-0.8476\varepsilon_{n-2}-0.0815\varepsilon_{n-3}$$

For *z*-axis raw data, the model identified is identified as ARMA(1,3):

(42) $$x_{n}=0.8593x_{n-1}+\varepsilon_{n}-0.51915\varepsilon_{n-1}+0.0328\varepsilon_{n-2}-0.0236\varepsilon_{n-3}$$

For *z*-axis BiLSTM network results, the model identified is identified as ARMA(3,3):

(43) $$x_{n}=2.0000x_{n-1}-1.2268x_{n-2}+0.2031x_{n-3}+\varepsilon_{n}-1.0446\varepsilon_{n-1}-0.0809\varepsilon_{n-2}-0.1086\varepsilon_{n-3}$$

where $x_{n}$ is the output of the ARMA model, $\varepsilon_{n}$ is the driving white noise (with mean, 0, and variance, ${\hat{\delta }}_{\varepsilon }^{2}$). The Kalman filter parameters are presented in Table 8. The value of R is the variance of the measurement. The initial value of the Kalman filter is set as follows: $x_{1}=\left[0;0;0;0\right]$, and $P_{1}$ is the fourth-order identity matrix.

#### 3.3.2. Estimating Kalman Filter Parameters Using the EM Algorithm

**Figure 8:**
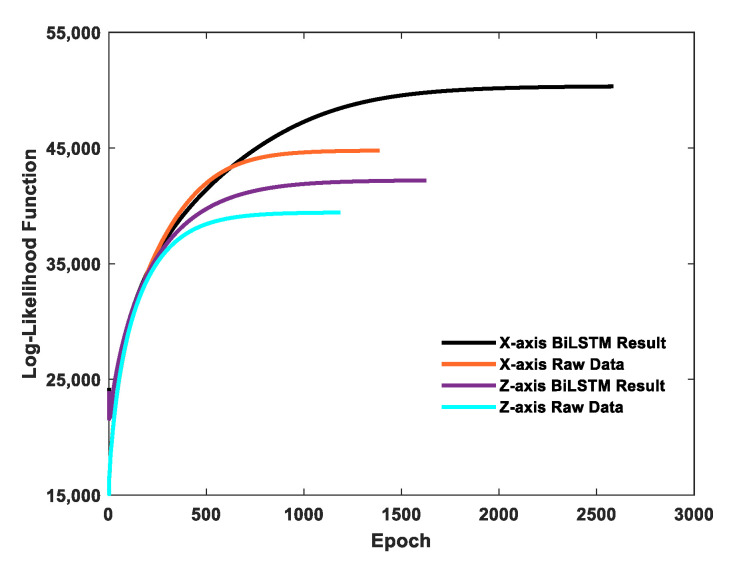
Log-likelihood value change of the iteration epoch.

When using the EM algorithm to estimate the Kalman filter parameters, only the iteration convergence conditions and initial parameters need to be set. The M-step convergence constant τ in Equation (22) was set to 0.1. The Kalman filter’s initial values were set to $x_{1}=0\;\mathrm{and}\;P_{1}=1$, and the Kalman filter’s initial parameters were set to $\Phi_{1}=1$, $H_{1}=1$, $Q_{1}=1$, and $R_{1}=1$. The change of the log-likelihood function during the iteration of the EM algorithm is shown in Figure 8. The parameter estimation results are presented in Table 9.

#### 3.3.3. Kalman Filtering Results

**Figure 9:**
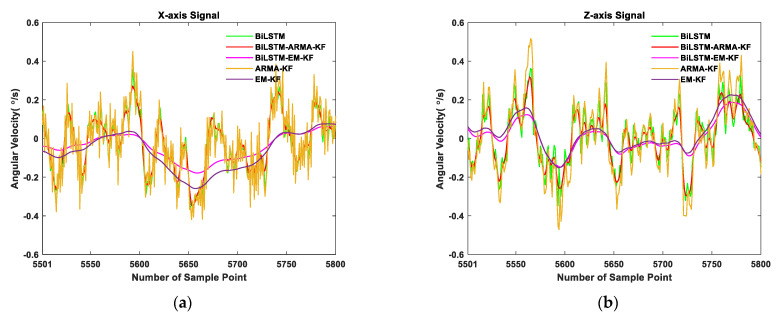
Comparison of error compensation performance of BiLSTM-EM-KF and BiLSTM-ARMA-KF. (**a**) Part of the *x*-axis data zoomed-in and (**b**) part of the *z*-axis data zoomed-in.

The results are illustrated in Table 10 and Table 11. For the *x*-axis data, the standard deviation of the BiLSTM-EM-KF results was reduced by 51.58% and 31.92% compared to the BiLSTM network and EM-KF, respectively. For the *z*-axis data, the standard deviation of the BiLSTM-EM-KF results was reduced by 29.19% and 12.75% compared to the BiLSTM network and EM-KF, respectively. Therefore, the combined method proposed in this paper can be demonstrated to improve the gyroscope error compensation performance of the BiLSTM network and EM algorithm. Moreover, compared with BiLSTM-ARMA-KF results, the standard deviation of the BiLSTM-EM-KF was reduced by 46.54% and 22.30% in *x*-axis and *z*-axis, respectively. It indicates the proposed method’s superior performance to that of BiLSTM-ARMA-KF. Furthermore, according to Figure 9, the curves of BiLSTM-EM-KF results are smoother, which proves that the proposed combined method is effective.

## 4. Conclusions

In this paper, a combined method of an LSTM network and Kalman filter is proposed for MEMS gyroscope error compensation in random vibration environments. Through the results, the following conclusions were obtained:

(1) After exploring proper input data step size and network topology, the network was trained, and the test results showed that the BiLSTM network outperformed the LSTM network, the GRU network, and the BiGRU network in gyroscope error compensation;
(2) Combining the BiLSTM network with the EM-KF method can improve their gyroscopic error compensation performance;
(3) In the classical gyroscope error compensation method, the ARMA-KF method, tedious data testing and model checking are required. In contrast, the EM-KF method only needs to set the initial parameters and the convergence value, which is much easier to apply. Moreover, the ARMA-KF method parameters cannot be updated through the filtering process, which means that satisfactory results cannot be obtained if the parameters are not defined correctly before the filtering process. From the filtering results, compared with BiLSTM-ARMA-KF, the standard deviation of the BiLSTM-EM-KF results were 46.54% and 22.30% lower, in *x*-axis and *z*-axis, and the output curve was smoother, which proves the effectiveness of the proposed method in this paper.

Future work should include conducting dynamic field experiments to obtain MEMS gyroscope outputs, as well as combining neural networks with more state-of-the-art Kalman filter methods for MEMS gyroscope error compensation and using fiber optic gyroscopes or laser gyroscopes as benchmarks for comparison.

## Figures and Tables

**Table 1:** Specifications of MSI320H gyroscope.

**GYRO**	Input range	±1800°/s
	Bias instability (Allan variance)	36°/h
	Angular random walk (Allan variance)	0.4°/√h
	Bandwidth (−3 dB)	≥220 Hz
**GENERAL**	Sample rate	100∼1000 Hz
	Weight	≤25 g
	Supply voltage	5.0 ± 0.5 V
	RS422 transmission bit rate	921600 bps
	Mechanical shock, any direction	≥20,000 g

**Table 2:** Specifications used for network training.

The output dimension of dense layer	1
Activation function of dense layer	ReLU
Dropout rate	0.5
Batch size	256
Training epoch	50
Learning rate	0.001

**Table 3:** Performance with varying values of the input data step.

Number of Hidden Layers	Number of Hidden Units	Input Data Step	STD (°/s)	Time/Epoch
10	64	5	0.1551	23 s
10	64	10	0.1481	38 s
10	64	15	0.1483	60 s
10	64	20	0.1346	82 s
10	64	25	0.1368	98 s
10	64	30	0.1501	115 s

**Table 4:** Performance with varying values of the number of hidden units.

Number of Hidden Layers	Number of Hidden Units	Input Data Step	STD (°/s)	Time/Epoch
10	8	20	0.1504	82 s
10	16	20	0.1459	82 s
10	32	20	0.1559	81 s
10	64	20	0.1346	82 s
10	128	20	0.1326	81 s
10	256	20	0.1468	88 s

**Table 5:** Performance with varying values of the number of hidden layers.

Number of Hidden Layers	Number of Hidden Units	Input Data Step	STD (°/s)	Time/Epoch
1	128	20	0.1493	10 s
2	128	20	0.1513	19 s
3	128	20	0.1597	27 s
4	128	20	0.1557	34 s
5	128	20	0.1658	42 s
6	128	20	0.1505	50 s
7	128	20	0.1470	58 s
8	128	20	0.1459	66 s
9	128	20	0.1542	75 s
10	128	20	0.1326	81 s
11	128	20	0.1405	90 s
12	128	20	0.1393	98 s

**Table 6:** Comparison of raw data, BiLSTM, LSTM, BiGRU, and GRU standard deviation values for *x*-axis data.

*x*-axis	STD (°/s)	Percentage
Raw data	0.2493	−
BiLSTM	0.1326	53.19%
LSTM	0.1543	61.89%
BiGRU	0.1501	60.21%
GRU	0.1604	64.34%

**Table 7:** Comparison of raw data, BiLSTM, LSTM, BiGRU, and GRU standard deviation values for *z*-axis data.

*z*-axis	STD (°/s)	Percentage
Raw data	0.2400	−
BiLSTM	0.1353	56.38%
LSTM	0.1550	64.58%
BiGRU	0.1504	62.67%
GRU	0.1574	65.58%

**Table 8:** Kalman filter parameters of all measurements.

	Φ	Γ	H	Q	R
*x*-axis raw data	$\left[\begin{array}{cccc} -0.3126 & 0.8168 & 0.1520 & 0\\ 1 & 0 & 0 & 0\\ 0 & 1 & 0 & 0\\ 0 & 0 & 1 & 0 \end{array}\right]$	$\left[\begin{array}{cccc} 1 & 0.6885 & -0.3241 & -0.0366\\ 0 & 0 & 0 & 0\\ 0 & 0 & 0 & 0\\ 0 & 0 & 0 & 0 \end{array}\right]$	$\left[\begin{array}{cccc} 1 & 0 & 0 & 0 \end{array}\right]$	$\left[\begin{array}{cccc} 0.0370 & 0 & 0 & 0\\ 0 & 0.0370 & 0 & 0\\ 0 & 0 & 0.0370 & 0\\ 0 & 0 & 0 & 0.0370 \end{array}\right]$	0.0604
*x*-axis BiLSTM	$\left[\begin{array}{cccc} 0.8505 & 0.8891 & -0.7745 & 0\\ 1 & 0 & 0 & 0\\ 0 & 1 & 0 & 0\\ 0 & 0 & 1 & 0 \end{array}\right]$	$\left[\begin{array}{cccc} 1 & 0.1354 & -0.8476 & -0.0815\\ 0 & 0 & 0 & 0\\ 0 & 0 & 0 & 0\\ 0 & 0 & 0 & 0 \end{array}\right]$	$\left[\begin{array}{cccc} 1 & 0 & 0 & 0 \end{array}\right]$	$\left[\begin{array}{cccc} 0.0036 & 0 & 0 & 0\\ 0 & 0.0036 & 0 & 0\\ 0 & 0 & 0.0036 & 0\\ 0 & 0 & 0 & 0.0036 \end{array}\right]$	0.0175
*z*-axis raw data	$\left[\begin{array}{cccc} 0.8593 & 0 & 0 & 0\\ 1 & 0 & 0 & 0\\ 0 & 1 & 0 & 0\\ 0 & 0 & 1 & 0 \end{array}\right]$	$\left[\begin{array}{cccc} 1 & -0.5191 & 0.0328 & 0.0236\\ 0 & 0 & 0 & 0\\ 0 & 0 & 0 & 0\\ 0 & 0 & 0 & 0 \end{array}\right]$	$\left[\begin{array}{cccc} 1 & 0 & 0 & 0 \end{array}\right]$	$\left[\begin{array}{cccc} 0.0405 & 0 & 0 & 0\\ 0 & 0.0405 & 0 & 0\\ 0 & 0 & 0.0405 & 0\\ 0 & 0 & 0 & 0.0405 \end{array}\right]$	0.0596
*z*-axis BiLSTM	$\left[\begin{array}{cccc} 2.0000 & -1.2268 & 0.2031 & 0\\ 1 & 0 & 0 & 0\\ 0 & 1 & 0 & 0\\ 0 & 0 & 1 & 0 \end{array}\right]$	$\left[\begin{array}{cccc} 1 & -1.0446 & 0.0809 & 0.1086\\ 0 & 0 & 0 & 0\\ 0 & 0 & 0 & 0\\ 0 & 0 & 0 & 0 \end{array}\right]$	$\left[\begin{array}{cccc} 1 & 0 & 0 & 0 \end{array}\right]$	$\left[\begin{array}{cccc} 0.0043 & 0 & 0 & 0\\ 0 & 0.0043 & 0 & 0\\ 0 & 0 & 0.0043 & 0\\ 0 & 0 & 0 & 0.0043 \end{array}\right]$	0.0183

**Table 9:** The parameter estimation results.

	Φ	H	Q	R
*x*-axis raw data	0.9723	0.1350	0.0016	0.1181
*x*-axis BiLSTM	0.9738	0.3422	0.0016	0.2852
*z*-axis raw data	0.9471	0.1327	0.0065	0.1155
*z*-axis BiLSTM	0.9530	0.2983	0.0044	0.2430

**Table 10:** Comparison of raw data, BiLSTM, ARMA-KF, EM-KF, BiLSTM-ARMA-KF, and BiLSTM-EM-KF standard deviation values for *x*-axis data.

*x*-axis	STD (°/s)	Percentage
Raw data	0.2493	−
BiLSTM	0.1326	53.19%
ARMA-KF	0.1618	64.90%
EM-KF	0.0943	37.83%
BiLSTM-ARMA-KF	0.1201	48.17%
BiLSTM-EM-KF	0.0642	25.75%

**Table 11:** Comparison of raw data, BiLSTM, ARMA-KF, EM-KF, BiLSTM-ARMA-KF, and BiLSTM-EM-KF standard deviation values for *z*-axis data.

*z*-axis	STD (°/s)	Percentage
Raw data	0.2400	−
BiLSTM	0.1353	56.38%
ARMA-KF	0.1853	77.21%
EM-KF	0.1098	45.75%
BiLSTM-ARMA-KF	0.1233	51.38%
BiLSTM-EM-KF	0.0958	39.92%

## Appendix A. Stationarity and Normality Tests

For stationarity, the most common test method is the run test. It was divided equally into 20 groups $\left\{X_{1},\;X_{2},\cdots ,X_{20}\right\}$, and the mean square value of each group was $\sigma_{i}$ where i∈[1,20]. The mean of $\left\{\sigma_{i}\right\}$ was $\sigma_{mean}$, and the difference between each $\sigma_{i}$ and $\sigma_{mean}$ formed $\left\{\sigma_{ai}\right\}$:

(A1) $$\sigma_{ai}=\sigma_{i}-\sigma_{mean}$$

The total number of positive values in $\left\{\sigma_{ai}\right\}$ was recorded as $n_{1}$, and the total number of negative values in $\left\{\sigma_{ai}\right\}$ was recorded as $n_{2}$. The number of positive and negative alternations in order and plus 1 more was the Run r. According to the run test table, whether r was within the confidence interval was determined at the significance level α=0.05.

After eliminating the trend term, the raw data and BiLSTM network results of the *x*-axis and *z*-axis met the stationarity requirements. The test process is presented in Table A1, Table A2, Table A3 and Table A4.

**Table A1:** Run test process of *x*-axis raw data.

$\sigma_{mean}=0.0604$ **,** $n_{1}=8$ **,** $n_{2}=12$ **,** r=14 **, Significance Level** α=0.05 **, Confidence Interval** [6,16] **.**										
	$X_{1}$	$X_{2}$	$X_{3}$	$X_{4}$	$X_{5}$	$X_{6}$	$X_{7}$	$X_{8}$	$X_{9}$	$X_{10}$
$\sigma_{i}$	0.0615	0.0583	0.0516	0.0621	0.0664	0.0572	0.0640	0.0545	0.0571	0.0628
$\sigma_{ai}$	0.0011	−0.0021	−0.0088	0.0017	0.0060	−0.0032	0.0036	−0.0059	−0.0033	0.0024
*r*	1	2		3		4	5	6		7
	$X_{11}$	$X_{12}$	$X_{13}$	$X_{14}$	$X_{15}$	$X_{16}$	$X_{17}$	$X_{18}$	$X_{19}$	$X_{20}$
$\sigma_{i}$	0.0601	0.0602	0.0565	0.0743	0.0585	0.0655	0.0532	0.0693	0.0588	0.0557
$\sigma_{ai}$	−0.0003	−0.0002	−0.0039	0.0139	−0.0019	0.0051	−0.0072	0.0089	−0.0016	−0.0047
r	8			9	10	11	12	13	14	

**Table A2:** Run test process of *x*-axis BiLSTM network results.

$\sigma_{mean}=0.0175$ **,** $n_{1}=11$ **,** $n_{2}=9$ **,** r=14 **, Significance Level** α=0.05 **, Confidence Interval** [6,16] **.**										
	$X_{1}$	$X_{2}$	$X_{3}$	$X_{4}$	$X_{5}$	$X_{6}$	$X_{7}$	$X_{8}$	$X_{9}$	$X_{10}$
$\sigma_{i}$	0.0189	0.0177	0.0144	0.0192	0.0198	0.0163	0.0191	0.0154	0.0159	0.0192
$\sigma_{ai}$	0.0014	0.0002	−0.0031	0.0017	0.0023	−0.0012	0.0016	−0.0022	−0.0016	0.0017
*r*	1		2	3		4	5	6		7
	$X_{11}$	$X_{12}$	$X_{13}$	$X_{14}$	$X_{15}$	$X_{16}$	$X_{17}$	$X_{18}$	$X_{19}$	$X_{20}$
$\sigma_{i}$	0.0176	0.0178	0.0159	0.0224	0.0149	0.0191	0.0144	0.0205	0.0166	0.0150
$\sigma_{ai}$	0.0001	0.0003	−0.0016	0.0049	−0.0026	0.0016	−0.0031	0.0030	−0.0009	−0.0025
r			8	9	10	11	12	13	14	

**Table A3:** Run test process of *z*-axis raw data.

$\sigma_{mean}=0.0596$ **,** $n_{1}=10$ **,** $n_{2}=10$ **,** r=10 **, Significance Level** α=0.05 **, Confidence Interval** [6,16] **.**										
	$X_{1}$	$X_{2}$	$X_{3}$	$X_{4}$	$X_{5}$	$X_{6}$	$X_{7}$	$X_{8}$	$X_{9}$	$X_{10}$
$\sigma_{i}$	0.0673	0.0565	0.0639	0.0694	0.0611	0.0570	0.0489	0.0533	0.0556	0.0539
$\sigma_{ai}$	0.0077	−0.0031	0.0043	0.0098	0.0015	−0.0026	−0.0107	−0.0063	−0.0040	−0.0057
*r*	1	2	3			4				
	$X_{11}$	$X_{12}$	$X_{13}$	$X_{14}$	$X_{15}$	$X_{16}$	$X_{17}$	$X_{18}$	$X_{19}$	$X_{20}$
$\sigma_{i}$	0.0577	0.0671	0.0642	0.0645	0.0585	0.0619	0.0516	0.0622	0.0620	0.0560
$\sigma_{ai}$	−0.0019	0.0075	0.0046	0.0049	−0.0011	0.0023	−0.0080	0.0026	0.0024	−0.0036
r		5			6	7	8	9		10

**Table A4:** Run test process of *z*-axis BiLSTM network results.

$\sigma_{mean}=0.0183$ **,** $n_{1}=9$ **,** $n_{2}=11$ **,** r=10 **, Significance Level** α=0.05 **, Confidence Interval** [6,16] **.**										
	$X_{1}$	$X_{2}$	$X_{3}$	$X_{4}$	$X_{5}$	$X_{6}$	$X_{7}$	$X_{8}$	$X_{9}$	$X_{10}$
$\sigma_{i}$	0.0216	0.0159	0.0200	0.0227	0.0180	0.0168	0.0139	0.0160	0.0158	0.0158
$\sigma_{ai}$	0.0033	−0.0024	0.0017	0.0044	−0.0003	−0.0015	−0.0044	−0.0023	−0.0025	−0.0025
*r*	1	2	3		4					
	$X_{11}$	$X_{12}$	$X_{13}$	$X_{14}$	$X_{15}$	$X_{16}$	$X_{17}$	$X_{18}$	$X_{19}$	$X_{20}$
$\sigma_{i}$	0.0173	0.0217	0.0213	0.0208	0.0175	0.0197	0.0154	0.0199	0.0191	0.0162
$\sigma_{ai}$	−0.0010	0.0034	0.0030	0.0025	−0.0008	0.0014	−0.0029	0.0016	0.0008	−0.0021
r		**5**			6	7	8	9		10

For normality, the most common test method is calculating skewness, ξ, and kurtosis, υ. When the calculated ξ is close to 0, and υ is close to 3, the data are considered to satisfy normality. For the valuation of *ξ* and *υ*:

(A2) $$\xi =E\left[{\left(\frac{x_{N}-e_{x}}{\sigma_{x}}\right)}^{3}\right]=\frac{E\left[{\left(x_{N}-e_{x}\right)}^{3}\right]}{{\left\{E\left[{\left(x_{N}-e_{x}\right)}^{2}\right]\right\}}^{\frac{3}{2}}}=\frac{\frac{1}{N}\sum_{n=1}^{N}{\left(x_{N}-e_{x}\right)}^{3}}{{\left[\frac{1}{N}\sum_{n=1}^{N}{\left(x_{N}-e_{x}\right)}^{2}\right]}^{\frac{3}{2}}}$$

(A3) $$\upsilon =E\left[{\left(\frac{x_{N}-e_{x}}{\sigma_{x}}\right)}^{4}\right]=\frac{E\left[{\left(x_{N}-e_{x}\right)}^{4}\right]}{{\left\{E\left[{\left(x_{N}-e_{x}\right)}^{2}\right]\right\}}^{2}}=\frac{\frac{1}{N}\sum_{n=1}^{N}{\left(x_{N}-e_{x}\right)}^{4}}{{\left[\frac{1}{N}\sum_{n=1}^{N}{\left(x_{N}-e_{x}\right)}^{2}\right]}^{2}}$$

where $e_{x}$ and $\sigma_{x}$ are the mean and standard deviation of the data, respectively.

After eliminating the trend term, the raw data and BiLSTM network results of the *x*-axis and *z*-axis met the normality requirements. The results are presented in Table A5.

**Table A5:** The normality test results.

	*x*-axis Raw Data	*x*-axis BiLSTM Network Results	*z*-axis Raw Data	*z*-axis BiLSTM Network Results
Skewness *ξ*	2.8329	2.7283	2.7294	2.8156
Kurtosis *υ*	0.0177	−0.1077	−0.0631	−0.0170

## Appendix B. Determine the ARMA Model Order

The Akaike information criterion can be expressed as follows:

(A4) $$\mathrm{AIC}\left(p,q\right)=N\ln\left(\delta^{2}\left(p,q\right)\right)+2\left(p+q+1\right)$$

where $\delta^{2}$ is the variance of driving white noise under each order and N is the total number of data samples. When AIC obtains the minimum value, the fitted model is the optimal model. In the case that the maximum values of p and q are set to 3, the AIC values at each order are shown in Table A6, Table A7, Table A8 and Table A9.

**Table A6:** AIC values of *x*-axis raw data at each order.

	*p* = 0	*p* = 1	*p* = 2	*p* = 3
***q* = 0**	−	−4350.7006	−5373.4026	−5483.9134
***q* = 1**	−2378.8661	−5480.4267	−5503.6485	−5503.2634
***q* = 2**	−3784.2642	−5501.9738	−5502.2446	−5501.9462
***q* = 3**	−4524.5464	−5504.1306	−5502.9798	−5514.9860

**Table A7:** AIC values of *x*-axis BiLSTM network results at each order.

	*p* = 0	*p* = 1	*p* = 2	*p* = 3
***q* = 0**	−	−33245.8540	−33390.6679	−33390.1658
***q* = 1**	−24407.6844	−33392.3127	−33390.3534	−33387.4822
***q* = 2**	−28837.8009	−33390.3543	−33391.2919	−33390.0297
***q* = 3**	−30742.3669	−33388.3649	−33390.0548	−33456.4704

**Table A8:** AIC values of *z*-axis raw data at each order.

	*p* = 0	*p* = 1	*p* = 2	*p* = 3
***q* = 0**	−	−3384.5655	−4241.9726	−4367.2696
***q* = 1**	−1992.4063	−4410.0138	−4414.9553	−4417.2529
***q* = 2**	−3107.6730	−4414.3979	−4414.5702	−4415.3240
***q* = 3**	−3612.4223	−4417.6140	−4415.7666	−4414.6351

**Table A9:** AIC values of *z*-axis BiLSTM network results at each order.

	*p* = 0	*p* = 1	*p* = 2	*p* = 3
***q* = 0**	−	−31059.1193	−31186.5670	−31212.5235
***q* = 1**	−23478.3934	−31198.8844	−31202.3767	−31212.2053
***q* = 2**	−27430.5011	−31206.1886	−31221.9048	−31228.0632
***q* = 3**	−29112.3646	−31212.9557	−31211.5881	−31262.0166

The first-order autocorrelation of the identified model residuals $\left\{\omega_{t}\right\}$ was tested by the Durbin–Watson method. Assuming that the first-order correlation of $\left\{\omega_{t}\right\}$ can be defined as:

(A5) $$\omega_{t}=\rho \omega_{t-1}+v_{t}$$

when ρ = 0, there is no first-order autocorrelation in $\left\{\omega_{t}\right\}$. Then the Durbin–Watson test value d:

(A6) $$d=\frac{\sum_{n=2}^{N}{\left(\omega_{n}-\omega_{n-1}\right)}^{2}}{\sum_{n=1}^{N}\omega_{n}^{2}}\approx 2\left(1-\rho \right)$$

The identified model’s Durbin–Watson test value is shown in Table A10, indicating that the estimated model satisfies the requirements.

**Table A10:** D-W test results.

	*x*-axis Raw Data	*x*-axis BiLSTM Network Results	*z*-axis Raw Data	*z*-axis BiLSTM Network Results
Durbin–Watson test value	1.9997	2.0064	1.9998	1.9903
